# Unraveling retrograde signaling pathways: finding candidate signaling molecules via metabolomics and systems biology driven approaches

**DOI:** 10.3389/fpls.2012.00267

**Published:** 2012-12-05

**Authors:** Camila Caldana, Alisdair R. Fernie, Lothar Willmitzer, Dirk Steinhauser

**Affiliations:** ^1^Brazilian Bioethanol Science and Technology Laboratory (Brazilian Center of Research in Energy and Materials)Campinas, Brazil; ^2^Department of Molecular Physiology, Max Planck Institute of Molecular Plant PhysiologyPotsdam-Golm, Germany

**Keywords:** retrograde signaling, metabolic signals, systems biology, metabolomics, sub-cellular metabolomics

## Abstract

A tight coordination of biological processes between cellular compartments and organelles is crucial for the survival of any eukaryotic organism. According to cellular requirements, signals can be generated within organelles, such as chloroplasts and mitochondria, modulating the nuclear gene expression in a process called retrograde signaling. Whilst many research efforts have been focused on dissecting retrograde signaling pathways using biochemical and genetics approaches, metabolomics and systems biology driven studies have illustrated their great potential for hypotheses generation and for dissecting signaling networks in a rather unbiased or untargeted fashion. Recently, integrative genomics approaches, in which correlation analysis has been applied on transcript and metabolite profiling data of *Arabidopsis thaliana*, revealed the identification of metabolites which are putatively acting as mediators of nuclear gene expression. Complimentary, the continuous technological developments in the field of metabolomics *per se* has further demonstrated its potential as a very suitable readout to unravel metabolite-mediated signaling processes. As foundation for these studies here we outline and discuss recent advances in elucidating retrograde signaling molecules and pathways with an emphasis on metabolomics and systems biology driven approaches.

## INTRODUCTION

Biological systems rely on complex interactions of heterogeneous small- and macro-molecules to execute cellular functions required for their growth, survival, and propagation (Smeekens et al., 2010). To maintain cellular homeostasis the interactions of and among these heterogeneous molecules need to be strictly regulated and coordinately modified to respond appropriately to external and internal stimuli. This fundamental process, which is common to all organisms, is broadly termed signal transduction. Generally, it involves the sensing of a stimulus or signal, the integration of information within and between different systems levels and finally, the execution of regulatory events resulting in a cellular response (Keurentjes et al., 2011; Baldazzi et al., 2012). While on a first glance signal transduction seems to be unidirectional with respect to information processing, a continuous sensing and control of the regulated function is required for fine-tuning.

In contrast to bacteria, the eukaryotic cell comprises a large number of diverse subcellular compartments and organelles which are usually delineated by a lipid bilayer to maintain specific microenvironments (Pogson et al., 2008; Krueger et al., 2011). Along with this many physiological processes and metabolic reactions are either solely localized in a single compartment or partitioned between them (Martinoia et al., 2007; Weber and Fischer, 2007; Linka and Weber, 2010). Although these compartments are physically and biochemically distinct, a tight coordination of processes between them is essential to maintain the biological functionality of the eukaryotic cell (Lunn, 2007).

While the different subcellular compartments are involved in storage, detoxification, and synthesis of a variety of specific and important compounds (Paris et al., 1996; Dyall et al., 2004; Becker, 2007), plastids and mitochondria in particular play also an essential role in integrating environmental cues into metabolic responses assisting in the adjustments of growth and development (Leon et al., 1998; Yang et al., 2008; Kessler and Schnell, 2009). However and in spite of the broad range of functions housed in, e.g., the plastids, only 5% of its proteome is encoded and synthesized by the plastids themselves (Abdallah et al., 2000). Chloroplasts and mitochondria are believed to have originated by endosymbiosis of free-living bacterial ancestors by a pre-eukaryotic cell, but contain (in comparison to their closest free-living relatives) only a strongly reduced portion of the ancestral genome with the majority of genes having been either lost or transferred to the nucleus (Andersson et al., 2003; Dyall et al., 2004). Given that many components of the energy transduction cascades are encoded by both the organelle and the nuclear genome, a tight coordination of gene expression in two or more cellular compartments is required to ensure the correct concentrations of organelle proteins, independent of their genesis, and thus to maintain organelle functions (Pogson et al., 2008; Kleine et al., 2009). Such genome-coordination mechanisms are achieved by bidirectional signaling that controls the information flux from the nucleus to the organelles (anterograde signaling; Sardiello et al., 2005) and by signals from the organelles to the nucleus (retrograde signaling; Biehl et al., 2005; Nott et al., 2006; Pesaresi et al., 2007; Jung and Chory, 2010). Retrograde signaling discloses the functional and developmental state of the organelles to the nucleus, which can then modulate anterograde control and cellular metabolism, as a result of environmental changes and signals perceived by the organelles (Woodson and Chory, 2008). Tight regulation of such signaling establishes the proper balance of gene expression and regulation in response to a fluctuating environment and thus, is fundamental for the survival of any organism. The intracellular communication between various organelles is quite complex and interdependent (Leister, 2005; Koussevitzky, 2007; Pesaresi et al., 2007; Jung and Chory, 2010). In photosynthetic eukaryotes, this scenario is even more complex owing the cross-talk between chloroplasts and mitochondria (Woodson and Chory, 2008).

## TARGETED AND BOTTOM-UP APPROACHES FOR SIGNAL MOLECULE VALIDATION

To date, an increasing but still small number of candidate signaling molecules and their respective pathways have been identified. So far, the putative sources of retrograde signals are thought to derive from (i) components of tetrapyrrole biosynthetic pathway intermediates (Mochizuki et al., 2001; Strand et al., 2003), (ii) reactive oxygen species levels in plastid (op den Camp et al., 2003; Wagner et al., 2004; Laloi et al., 2006; Lee et al., 2007), (iii) the redox state of organelles (Bonardi et al., 2005; Piippo et al., 2006; Pesaresi et al., 2007), and (iv) protein synthesis (Koussevitzky, 2007; for recent reviews, see Leister, 2005, 2012; Nott et al., 2006; Koussevitzky, 2007; Pesaresi et al., 2007; Pogson et al., 2008; Kleine et al., 2009; Chan et al., 2010; Jung and Chory, 2010; Pfannschmidt, 2010; Leister et al., 2011). However, most of the research on retrograde signaling has targeted isolated pathways under artificial and/or pleiotropic conditions (Kleine et al., 2009). One of the best-studied chloroplast retrograde signaling pathways, the tetrapyrrole pathway provides an example of the complexity involved in unequivocally identifying a retrograde signal. Photo-oxidative damage of undeveloped chloroplasts leads to the accumulation of Mg-proto, an intermediate metabolite of chlorophyll biosynthesis, which represses nuclear genes encoding for photosynthesis-related proteins (Strand et al., 2003). Genetic screenings associated to microarray analysis in *Arabidopsis* have resulted in identifying the *genomes uncoupled* (*gun*) mutants, which block Mg-proto mediated retrograde signaling by affecting its accumulation (Mochizuki et al., 2001; Strand et al., 2003; Koussevitzky, 2007). Those earlier studies have found a direct correlation of nuclear gene expression and total cellular levels of Mg-proto IX estimated using fluorescence spectrometry (Mochizuki et al., 2001). However, later studies that employed a more sensitive and reproducible HPLC methodology revealed the absence of any correlation between Mg-proto IX and nuclear gene expression levels (Mochizuki et al., 2008). These finding argues against the proposed model for Mg-proto IX as a retrograde signal. Thus, further investigations using alternative analytical approaches together with subcellular localization estimation might be necessary to unambiguously accept or reject the involvement of Mg-proto IX in retrograde signaling. Therefore, the unambiguous experimental validation of a compound as a signaling molecule is an extremely laborious task and so far, arguably no “true” signaling molecule leaving the plastid has been identified (Pfannschmidt, 2010). Innovative biochemical and genetic screenings, such as inducible expression or inducible RNA interference (RNAi) are supposed to aid in further identifying proteins and signals in these pathways (Woodson and Chory, 2008). As the function of a cellular constitute might also be context-dependent, more holistic ’omics-like approaches might be helpful in elucidating the specificity of a candidate signaling molecule.

## IDENTIFICATION AND VALIDATION OF NOVEL SIGNALING CANDIDATES VIA METABOLOMICS

Whilst much research efforts have been focused on dissecting signaling pathways using biochemical and genetics approaches, the number of yet discovered mechanisms of retrograde signaling are likely not sufficient to explain the tight regulation and interdependence of organelle functions. For instance, the cellular metabolism is tightly correlated with processes occurring in chloroplasts and mitochondria. It is widely believed that changes in the concentration of metabolites triggered by the organelles are likely sensed in the cytosol and thus, might regulate nuclear gene expression (Kleine et al., 2009). Therefore, metabolites are thought to be the most likely candidates for retrograde signaling molecules, since the metabolites exported from the organelles into the cytosol are likely reflective of the organellar metabolic state (Leister, 2012). A consequence of this view is that fluxes of metabolites between, e.g., the chloroplast and the cytosol can mediate information on the chloroplast redox stage and therefore, acting as potential intracellular signals (Kleine et al., 2009). For instance, trehalose 6-phosphate (T6P) promotes thioredoxin-mediated redox transfer to AGPase in response to cytosolic sugar levels, reporting on the metabolic status between the cytosol and the chloroplast (Kolbe et al., 2005). Another example of alteration in the cellular redox status has been reported for malate in tomato fruits (Centeno et al., 2011).

Over the past decades a number of analytical methods, such as gas chromatography (GC), liquid chromatography (LC), capillary electrophoresis (CE) coupled to mass spectrometry (MS) or nuclear magnetic resonance (NMR), have been developed and improved to accurately and sensitively analyze small molecules from complex sample mixtures (Kopka et al., 2004; Lisec et al., 2008; Lei et al., 2011). As these technologies enable the simultaneous detection of several hundred to several thousand metabolites, a more holistic view of cellular functions can be gathered since metabolites are considered to directly reflect the physiological status of a cell. Therefore, metabolite profiling has been considered as a powerful tool for the unbiased ability to characterize and differentiate genotypes and phenotypes (Hirai et al., 2005; Kusano et al., 2007; Hannah et al., 2010; Caldana et al., 2011) and provides also an excellent readout for the dissection of novel signaling pathways. Unfortunately, the measurement of all metabolites using a single analytical technology is yet not feasible due to the vast number of compounds which on top differ widely in their chemical properties, such as size, polarity, stability, and quantity, as well as in their immense dynamic concentration ranges (Kopka et al., 2004; Lisec et al., 2008; Lei et al., 2011). The optimal selection of a technology will thus largely depend on the study aim and is usually a compromise of selectivity, speed, and throughput (Lei et al., 2011; Krueger et al., 2012). Therefore, orthogonal analytical technologies in conjunction with targeted and untargeted data analyses will support a broader compound coverage as the chemical properties and also the structure of molecules involved in signaling might not be known to date. Nonetheless, some metabolites might still not be precisely captured using a particular analytical ’omics technology or their combinations, thus requiring targeted biochemical assays either simultaneously applied or used for verification.

Several lines of evidence suggested that the development of target methods with high accuracy is very informative in elucidating the role of candidate signals. One successful example of the use of metabolic profiling for elucidating retrograde signals in plants is the identification of methylerythritol cyclodiphosphate (MEcPP; Xiao et al., 2012). Using a genetic approach the authors identified the retrograde signaling *ceh1* mutant, which displayed changes in expression of stress-related genes associated to salicylic acid response and resistance to the pathogen *Pseudomonas*. *Ceh1* is caused by a mutation in the *HDS* gene responsible for the conversion of MEcPP to hydroxymethylbutenyl diphosphate (HMBPP) in the methylerythritol phosphate (MEP) pathway. Metabolite profile analysis of MEP pathway intermediates by LC-MS revealed an accumulation of the intermediate metabolite MEcPP in *ceh1.* MEcPP is a specific and critical retrograde signaling metabolite that acts as a stress sensor by triggering the expression of specific stress-responsive nuclear encoded plastidial proteins (Xiao et al., 2012).

## IDENTIFICATION AND VALIDATION OF NOVEL SIGNALING CANDIDATES VIA SUB-/CELLULAR METABOLOMICS

Despite the great potential of metabolomics in identifying novel signals, the majority of studies rely on the entire set of metabolic reactions that can take place within different tissues, but do not consider the type of tissue or the subcellular specificity and localization of metabolites. Taking this observation into account, only metabolites whose changes are readily transferable between compartments represent promising retrograde signals (Kleine et al., 2009; Leister, 2012). Thus, unraveling the subcellular localization of metabolites and their dynamics are crucial for identifying small molecules within organelles that potentially trigger retrograde signaling. The main challenge of such analysis is the fast conversion and reallocation of metabolites out of organelles. To date, several methods have been developed to monitor the spatial distribution of metabolites within the different cell types and cellular compartments (for a review, see Krueger et al., 2012).

Protoplast fractionation has been widely used to quantify metabolite levels in purified organelles such as chloroplasts, mitochondria, and the vacuole, respectively (e.g., Robinson and Walker, 1980; Stitt et al., 1983; Gerhardt and Heldt, 1984; Dancer et al., 1990; Martinoia et al., 1991; Gardestrom, 1993; Abdallah et al., 2000; Tohge et al., 2011). However, the procedure is very time-consuming as it includes several centrifugation steps, therefore causing a disturbance of the physiological and biochemical system (Krueger et al., 2012). Consequently, such artificial system may not accurately reflect the *in planta* situation. Recently, protoplast fractionation was used to detect the subcellular levels of 3′- Phosphoadenosine 5′-phosphate (PAP) and confirm its role as a retrograde signal (Estavillo et al., 2011). PAP was found to accumulate in response to drought and high light and is regulated by the enzyme SAL1, which is present in chloroplasts and mitochondria (Estavillo et al., 2011). The cellular levels of PAP correlated well with the nuclear gene expression. Interestingly, transgenic targeting of SAL1 to either the nucleus or chloroplast of *sal1* mutants reduced the total PAP levels (Estavillo et al., 2011). However, except for the chloroplast, the subcellular quantification of PAP fractions has failed due to technical reasons (Estavillo et al., 2011; Leister, 2012).

A more accurate technique to monitor spatial and temporal metabolic changes in cellular compartments of intact tissues is the use of genetically encoded metabolite nanosensors. The fluorescence resonance energy transfer (FRET) nanosensor makes use of a recognition element (a protein that binds with the metabolite of interest) fused to a report element (a fluorophore pair). Changes in protein conformation triggered by ligand (metabolite)-recognition element binding leads to the emission of fluorescent light via the report element (for review, see Frommer et al., 2009). In *Arabidopsis*, FRET-based glucose and sucrose sensors have been used to successfully monitor sucrose and glucose transport in root tips (Chaudhuri et al., 2008, 2011). To follow the dynamic changes of a given metabolite with subcellular resolution, the FRET sensor has to be flanked with a signal sequence recognized by the organelle (e.g., nuclear or ER signal sequence) to enable the proper import of the sensor into the organelle (Hou et al., 2011). As each metabolite however requires its own sensor, it remains an arduous task to engineer sensors for various potential signaling molecules. Thus, only small molecules with previous strong evidence of being a putative signal can likely be considered in such an approach.

The probably most promising strategy to analyze subcellular metabolite distribution is the non-aqueous fractionation method which separates cellular compartments under conditions where metabolite translocation and conversion are completely arrested (Gerhardt and Heldt, 1984; Riens et al., 1991). It has a proven record especially in plant science (Gerhardt et al., 1987; Riens et al., 1991; Winter et al., 1993; Fettke et al., 2005; Krueger et al., 2009) and routinely facilitates the separation of three distinct compartments from each other, namely the cytosol, the plastids, and the vacuole (Gerhardt et al., 1987; Farre et al., 2001; Krueger et al., 2009, 2011). Although NAF produces continuous metabolite distributions of organelles and a successful separation of mitochondria is not yet reported, the entire cellular content and thus its metabolites are represented in the gradient which can be considered as an advantage for identifying signaling molecules (Jung and Chory, 2010). As the collected NAF fractions can be analyzed with modern high-throughput metabolite profiling techniques several hundred molecules from different broad compound classes can be measured simultaneously (Krueger et al., 2011) and finally assigned into compartments using improved statistical tools (Klie et al., 2011). Thus, the impact of environmental changes on cellular and subcellular metabolism and the cell’s state can be analyzed with high spatial resolution providing the necessary basis to discover novel signaling molecules. Recently, non-aqueous fractionation (NAF) in conjunction with orthogonal metabolite profiling technologies was applied to unravel the subcellular localization of primary and secondary metabolites as well as lipids (Krueger et al., 2011). While this study depicts the subcellular localization for a large number of chemically diverse metabolites, it also illustrates that there is the potential to further increase the compartmental resolution as various metabolites could not be unambiguously assigned into one of the three resolved compartments.

## SYSTEMS BIOLOGY APPROACHES IN RETROGRADE SIGNALING

The complexity and interdependence of the pathways involved in intracellular communication have a dramatic impact on cellular levels. To understand how plants adjust their biochemical machineries on several levels of biological information, a more holistic approach such as systems biology is required. Recent advances of high-throughput technologies and analytical methods, such as transcriptomics and metabolomics, have supported multilevel phenotyping and can help to understand many complex biological processes by generation of hypothesis about the dynamic system (Jung and Chory, 2010). Application of such systems-orientated analyses in retrograde control may provide novel means to unravel intracellular communication and corresponding signaling molecules.

Gene expression profiling has been widely used to study retrograde control. The classical approach to identify a set of nuclear genes under retrograde control is based on transcriptomics analysis of mutants defective in retrograde pathways (for examples, see the reviews of Jung and Chory, 2010). However, to avoid biased results caused by pleiotropic effects of a single experiment, it is highly recommended to use expression profile data of multiple experimental conditions. As an example, meta-analysis of 11 microarray experiments from mutants (e.g., *aox1a* and *msd1*-RNAi, among others) and short-term chemical treatments (e.g., antimycin A and rotenone) involved in plant mitochondrial dysfunction has been carried out to identify targets and pathways involved in mitochondrial retrograde signaling (Schwarzlander et al., 2012). Regardless of the level of severity of these mitochondrial impairments, three main retrograde signaling targets were identified, namely protein synthesis, photosynthetic light reactions, and plant–pathogen interactions (Schwarzlander et al., 2012). A similar approach has been performed to unravel intra- and inter-compartmental transcriptional networks coordinate the expression of genes for organellar functions (Leister et al., 2011).

Another recent study investigated the time-dependent impact of redox signals at both transcriptome and metabolome levels of *Arabidopsis thaliana* growing under either PSI or PSII light (Brautigam et al., 2009). The authors showed rapid and dynamic changes in nuclear transcript accumulation, which resulted in differential expression pattern for genes associated with photosynthesis and metabolism (Brautigam et al., 2009). This work proposed that photosynthesis acts as an environmental sensor, producing redox signals that perform a fine-tuning not only for photosynthesis but also for metabolic reactions (Brautigam et al., 2009).

Integration of high-throughput transcript and metabolite data has facilitated the identification of small molecules as potential mediators of gene expression (Hannah et al., 2010). Extensive analysis of transcript and metabolite correlations across a wide ranging of environmental conditions revealed single, highly-connected metabolites which correlated with several hundred to thousand transcripts. Among the candidates, compounds derived from carotenoid metabolism such as cryptoxanthin, zeaxanthin, and lutein were found to significantly correlate with a large number of transcripts. Interestingly, those metabolites displayed a significant overlap between positively correlated genes and those repressed by norflurazon, a known inhibitor of phytoene desaturase in the carotenoid biosynthesis (Hannah et al., 2010).

Another important issue for the identification of signals is to distinguish between “true” primary targets from tertiary targets (Pfannschmidt, 2010). Several lines of evidence demonstrated that retrograde signal may affect nuclear gene expression in a fast and dynamic range. Therefore, a combination of high resolution kinetic analysis combined with inducible systems and subcellular metabolite flux would allow setting a signal at a given time point. A recent example of this is that of *O*-acetylserine (OAS) which has been recently identified as a regulator of the sulfur status in *Arabidopsis* using computational analysis of time-series experiments and inducible transgenic plants revealing conditional increased OAS levels (Hubberten et al., 2012). However, it is important to note that OAS can be synthesized in multiple subcellular compartments. So, it is highly unlikely to represent a retrograde signal since such signals should confer organelle specific information. This is a crucial aspect of retrograde signaling which due to the extensive compartmentation (and three genomes) of the plant cell renders the identification of non-redundant signals highly complex. However, it would appear likely that the spatial-temporal resolution of metabolite levels and metabolite exchange between organelles will greatly aid in the detection and ultimately the mechanistic understanding of retrograde and anterograde signaling orchestrating plant organellar and nuclear gene expression.

## Conflict of Interest Statement

The authors declare that the research was conducted in the absence of any commercial or financial relationships that could be construed as a potential conflict of interest.
